# Medicine 4.0: the era of revolutionary access

**DOI:** 10.3389/fmed.2026.1903811

**Published:** 2026-07-27

**Authors:** Chris Macdonald

**Affiliations:** University of Cambridge, Cambridge, United Kingdom

**Keywords:** digital health, healthcare access, healthspan, lifespan, Medicine 3.0, Medicine 4.0, personalised medicine, preventive medicine

## Abstract

The evolution of modern healthcare is transitioning from a reactive, disease-centered paradigm (Medicine 2.0) toward a proactive, personalised model focused on maximising healthspan (Medicine 3.0). While this shift represents a vital optimisation of medical philosophy, it remains critically incomplete. This Perspective proposes a new paradigm (Medicine 4.0) which adds a foundational third dimension to the lifespan-healthspan model: radical access to both medical services and scientific ideas. The author analyses how commercial incentives, regulatory frameworks, and market forces frequently distort the production of scientific evidence, marginalise transformative, low-cost treatments, and delay paradigm shifts. Furthermore, the author examines the dual-edged nature of emerging digital health technologies, warning that monetisation through paywalls and engagement-driven ad models risks ossifying existing public health inequalities rather than democratising care. By realigning institutional incentives and dismantling structural obstructions to inquiry, Medicine 4.0 advocates for a return to the foundational Baconian principle that scientific knowledge must serve humanity. Ultimately, the author argues that medical progress must be evaluated not merely by its technological surface area, but by its total societal volume.

## Introduction

Medicine is evolving at such a pace that it is difficult to keep up. What version are we at now? Can medicine even be meaningfully numbered, given that the scientific progress is inherently iterative and continuous? While any attempt to categorise medicine into distinct ‘versions’ risks oversimplifying a complex process, the three broad phases proposed by Attia and Gifford (1) in their book, *Outlive*, offer a valuable perspective. Their three phases of *Medicine 1.0, 2.0, and 3.0* provide a useful framework for thinking about how medicine has evolved and how it should evolve in the future.

*Medicine 1.0* is defined as the largely ineffective medicine practised before the emergence of modern science. During this time, treatments were often based on superstition, anecdote, or incomplete theories of the body, with limited ability to reliably produce optimal results.

*Medicine 2.0*, by contrast, represents the modern era of scientific medicine and evidence-based healthcare. Defined by the use of the scientific method and scientific tools such as microscopes, antibiotics, and randomised controlled trials. As Attia and Gifford state, “*Medicine 2.0* was transformational”; it became “a scientific war machine that has eradicated deadly diseases such as polio and smallpox,” contributing to the doubling of lifespans (2023). However, Attia and Gifford also argue that lifespans have since plateaued and modern healthcare systems are largely reactive (2023).

To be clear, Attia and Gifford are not suggesting abandoning *Medicine 2.0*. Rather, they advocate transitioning towards what they call *Medicine 3.0* which has four main points:

1. A far greater emphasis on prevention.
2. A better consideration of the patient as a unique individual.
3. A better consideration of risks (including the risk of doing nothing).
4. A far greater emphasis on healthspan and quality of life.

Accordingly, *Medicine 3.0* can be understood not as a replacement for modern medicine, but as an expansion of its remit. In particular it seeks to expand the intervention window earlier, and it seeks to better encompass long-term quality of life.

Both key aspects are logical and, from my perspective, very welcome upgrades. With regard to prevention, you could reframe it as merely being more efficient; why spend hours carefully removing a deadly tumour when you might be able to stop it from ever forming in the first place? Likewise, as we are now afforded a considerably longer life, it makes sense that we pay more attention to the quality of that life. Furthermore, a focus on quality of life allows for greater ambition for future healthcare. We can significantly upgrade human potential far above merely preventing ill health. As Attia and Gifford note, “We want more out of life than simply the absence of sickness or disability. We want to be thriving” (2023).

Put simply, you could think of *Medicine 3.0* as a logical response to the evolution of the primary causes of death. As Attia and Gifford note, once advances such as germ theory, sanitation, and antibiotics became established, surviving childbirth and childhood became increasingly normal, allowing people to live long enough to face more distant conditions such as colon cancer and Alzheimer’s disease (2023). As the event horizon of death moved further away, the opportunity is presented to shift from reaction to prevention.

While I welcome and encourage the transition to *Medicine 3.0*, for me there is something critical missing. A key consideration that would have a profoundly positive impact. In what I call *Medicine 4.0*, I keep the aforementioned upgrades but include a focus on radically improving access. Both access to ideas and access to services.

**Figure 1:**
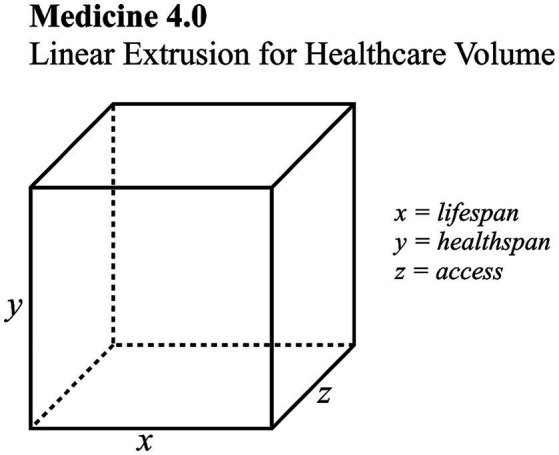
The axes of Medicine 4.0.

If we were to plot healthspan on the *y-axis* (quality of life), and lifespan on the *x-axis* (duration of life), then we could think of access as an additional dimension on the z-axis (visualised in Figure 1). An uncompromising commitment to expanding the depth of the resulting cuboid means, from a philosophical stance, that we believe that no idea should be left unexplored, and above all, no person should be left behind. The model marks a perspective shift from surface, to surface and volume.

It is important to emphasise that the *Medicine 4.0* concept proposed here is a direct philosophical extension of Attia and Gifford’s *Medicine 3.0* framework, rather than an alignment with other technological “*4.0*” paradigms (e.g., *Industry 4.0, Healthcare 4.0, Web 4.0*). Furthermore, it must be clearly delineated from contemporary frameworks like *Precision Medicine* or *Value-Based Healthcare*. While the latter has successfully shifted reimbursement models toward patient outcomes, it remains an operational framework that optimises care for those already within the system. It does not fundamentally challenge the systemic forces suppressing disruptive scientific inquiry, nor does it resolve the exclusion of disadvantaged populations from foundational health infrastructure. *Medicine 4.0* uniquely brings these paradigms together to form a dual mandate to democratise both access to healthcare and access to knowledge itself. To conceptualise this: *Medicine 4.0* is not simply about better treatment or better prevention. It is about building a healthcare system that is more open, more accessible, and fundamentally more scientific. It is best understood not merely as an economic or technological framework, but as a normative philosophy centred on maximising the total societal and scientific volume of medical progress.

### Access to services

The uncomfortable reality is that many of the world’s most effective preventative tools and treatments already exist. The problem is not always scientific capability, but access.

In wealthy nations, preventative medicine is often positioned as a premium product. Early diagnostics, specialist testing, continuous health monitoring, and personalised care are often available primarily to those who can afford private healthcare or expensive insurance.

Globally, the disparity is even sharper. Entire regions of the Global South still lack reliable access to basic medicines, vaccinations, clean water, and primary healthcare infrastructure. Conversations about optimising healthspan can sound almost surreal in places where preventable diseases remain daily realities (2–5).

This is not an argument against innovation. Quite the opposite. Medical progress should accelerate. But progress that cannot reach people ceases to be progress in any meaningful societal sense. A medicine available only to elites is not a healthcare revolution; it is a market advantage. It only increases inequalities.

In *Medicine 4.0*, accessibility is foundational. Lifespan and healthspan should not be determined by postcode, income bracket, or geopolitical luck. Medicine for all must become a non-negotiable standard. We should be ambitious and bold in this endeavour. Not merely getting better at inclusive practices and accessible design, but working towards a world where excellent healthcare is free at the point of delivery for all.

### Access to ideas

Access problems extend beyond services into something even more fundamental: access to scientific exploration itself.

Medical history is often presented as an inevitable march toward truth, as though every promising idea naturally rises to the surface. Reality is far messier. Scientific inquiry is shaped by regulation, funding structures, cultural stigma, politics, and commercial incentives. Entire fields can remain dormant for decades not because they lack potential, but because complex systems can discourage or even ban their exploration.

Few examples illustrate this more clearly than psychedelics. For decades serious medical research into compounds such as psilocybin and ibogaine was largely frozen across much of the Western world.

The 1970 Controlled Substances Act marked a critical turning point for psychedelic research, establishing severe regulatory barriers by classifying these substances as Schedule I (defined as having a high potential for abuse and no accepted medical use). While the ban effectively halted research under the guise of public health, historical evidence reveals this prohibition was fundamentally a weapon of political suppression enacted by an administration deeply entwined with the military-industrial complex. As admitted by former domestic policy advisor John Ehrlichman, the Nixon White House strategically criminalised substances associated with the anti-war and civil rights movements to disrupt their organising efforts, raid their homes, and vilify their leaders (6, 7). This political strategy protected an administration heavily invested in the Vietnam War effort, a conflict sustained by immense, often illicit, financial contributions from massive defence contractors seeking to preserve their lucrative government contracts (8, 9). By weaponising drug laws, the administration systematically neutralised its most vocal critics while safeguarding the economic interests of the defence sector that helped bankroll the presidency.

Adding to the barriers imposed by state prohibition, critics contend the pharmaceutical industry systemically suppressed the field; short-term, naturally occurring treatments posed a direct economic threat to the highly lucrative, daily medication model of, for example, conventional antidepressants (10, 11). Thus, the field faced a double barrier of political and corporate constraint. Accordingly, leading academics such as Psychiatrist Stanislav Grof argued that the decline of psychedelic research was driven more by external pressures than by scientific evidence demonstrating a lack of therapeutic value (12). Indeed, it is telling that when researchers finally resumed human clinical trials in the early 1990s, pioneers like Dr. Rick Strassman were only able to secure regulatory approval by explicitly framing their studies around investigating the drugs’ potential harms, toxicity, and psychosis-mimicking effects, carefully omitting any mention of therapeutic promise (13). In short, the pioneers had to adopt creative workarounds rather than being proactively supported by an optimised system.

Today, a resurgence of rigorous clinical research is demonstrating the remarkable therapeutic potential of psychedelics, which, in several instances are surpassing the efficacy of conventional long-term pharmaceutical approaches. Recent Phase 3 clinical trials published in *Nature Medicine* have shown that MDMA-assisted therapy produces profound, clinically significant reductions in severe PTSD symptoms, with over two-thirds of participants no longer meeting the diagnostic criteria for the disorder following treatment (14). Similarly, landmark randomised controlled trials have highlighted the rapid and sustained antidepressant effects of psilocybin for both major depressive disorder and treatment-resistant depression, often achieving remission where standard selective serotonin reuptake inhibitors (SSRIs) have failed (15, 16). Furthermore, compelling evidence demonstrates psilocybin’s robust efficacy in drastically reducing heavy drinking days among patients with severe alcohol use disorder (17), as well as providing enduring relief from existential distress and end-of-life anxiety in terminally ill cancer patients (18). By targeting underlying psychological trauma and catalysing rapid neural plasticity, these compounds are actively shifting the psychiatric paradigm away from chronic daily symptom management toward short-term, curative-focused interventions.

This naturally raises uncomfortable questions. How many other promising treatments are delayed, marginalised, suppressed, or ignored because they conflict with prevailing social norms or political or economic interests?

This is not a simplistic argument that pharmaceutical companies are maliciously ‘keeping us ill.’ Modern medicine has produced extraordinary breakthroughs. But incentive structures matter. For example, systems rewarded for managing chronic illness will not always align perfectly with low-cost interventions capable of producing transformative outcomes after limited or even single-use.

Crucially, however, we must distinguish these systemic obstructions from the necessary, functional delays inherent to modern healthcare. The rigorous demands of clinical trials, pharmacovigilance, cost-effectiveness assessments, and the establishment of new medical infrastructure are vital components of clinical validation and rollout. While these mechanisms inherently slow the translation of innovation into practice, they represent essential due process designed to ensure patient safety and therapeutic efficacy. Medicine 4.0 advocates for improving the efficiency of these processes where possible, but does not advocate for bypassing vital rigour. The mandate is to ensure that the initial scientific inquiry remains open. The objective is to guarantee that the ideas entering this rigorous validation process are elevated based on their scientific merit and potential to maximize human health, rather than being filtered out prematurely simply because they are, for example, politically inconvenient.

Medicine evolves within markets, institutions, regulatory systems, and shareholder expectations. These forces inevitably shape which ideas receive funding, which treatments are approved, and which possibilities remain unexplored.

### Incentive structures

This leads to perhaps the most important question of all: what exactly is modern healthcare incentivised to optimise?

Healthcare systems, like all systems, respond to incentives with consistency. When hospitals are funded by treatment volume, treatment increases, a phenomenon long described in health economics as supplier-induced demand (19, 20). When pharmaceutical profitability depends on long-term consumption, chronic management models tend to dominate. And when prevention generates less immediate financial return than intervention, prevention often struggles for priority (21).

Systems do not need villains to produce distorted outcomes; they simply require incentives misaligned with long-term human wellbeing.

The most successful forms of prevention are invisible. A tumour that never forms, a heart attack that never occurs, or a neurodegenerative disease delayed by 20 years often produces no clear dramatic intervention, no billable procedure, and no immediate signal of success, yet may represent medicine at its most powerful (22).

In multiple industries, strong incentives have been shown to shape the production and interpretation of evidence itself. Tobacco companies such as Philip Morris International funded research and messaging campaigns that emphasised uncertainty around the harms of smoking, delaying regulatory action for decades (23, 24). The sugar industry, through the Sugar Research Foundation, played a documented role in shaping mid-20th-century nutritional science by minimising the role of sugar in cardiovascular disease (25). In pharmaceuticals, companies such as Purdue Pharma have faced legal findings for misrepresenting evidence on opioid addiction risk, contributing to widespread patterns of long-term prescribing (26, 27).

Scientific attention is not evenly distributed by truth value alone. It is filtered through regulatory environments, funding availability, reputational risk, and commercial incentives (28, 29).

If we want the best possible healthcare systems, we must also understand and, where necessary, redesign the incentive structures that determine which ideas are explored, which are neglected, and which are ultimately translated into practice (21). We must consider the aforementioned *z-axis.* We must expand and defend the field on which the gift of the scientific method can perform and deliver.

### Digital health

As digital health tools, apps, and AI-driven interventions become more widespread, they offer a major opportunity to expand access to care at relatively low marginal cost. Such tools could significantly reduce barriers and support preventative medicine at scale. Data tracking and preventative action could become a matter of routine and what works in one location could be instantly deployed in remote and less developed areas.

Therefore, as we begin the rollout of such tools, we should remember that we do not have to monetise and privatise them. Paywalls and subscription models can exclude those who need help the most, and risk exacerbating and ossifying existing health inequalities. What could become genuinely scalable public health infrastructure can easily become another tiered system of access based on ability to pay.

Similarly, ad-supported models introduce incentives that may not align with real-world wellbeing. When revenue is driven by engagement or retention, developers may begin to optimise for attention and screen time rather than beneficial patient outcomes. Such systems can thrive on suppression and long-term perpetual dependence.

In essence, as more options become available, we should seek to learn from mistakes made previously. We should embrace this as an opportunity to be wise. To do things differently. To set new standards. To apply *Medicine 4.0*.

## Conclusion: returning to great science

In the 1600s, Sir Francis Bacon argued that knowledge should serve humanity, not as an abstract intellectual exercise, but as a practical tool for improving the human condition through observation, experimentation, and continual revision (30–32). To many, this was the birth of the scientific method as we know it today (33–36).

At the same time, Bacon was deeply concerned with that which might obstruct that goal. In his framework of the *Idols of the Tribe, Cave, Marketplace, and Theatre*, he described the systematic ways human understanding becomes distorted or restrained: by innate cognitive bias, personal perspective, language and communication, and the authority of systems and organisations (31). In short, medicine does not evolve in a vacuum and the scientific method is not inherently protected. It is not always permitted to serve its greatest function as a practical tool that improves the human condition.

*Medicine 4.0* is not simply about better treatment or better prevention. It is about building a healthcare system that is more open, more accessible, and fundamentally more scientific. It is about removing the barriers in the way of great science. It is about building a better system that not only empowers us to live better lives but also to become better people.

## Data Availability

The original contributions presented in the study are included in the article/supplementary material, further inquiries can be directed to the corresponding author/s.
